# Happy to Be a Boss? Cultural Moderators of Relationships Between Supervisory Responsibility and Job Satisfaction

**DOI:** 10.3389/fpsyg.2022.868910

**Published:** 2022-06-15

**Authors:** Krista Jaakson, Gaygysyz Ashyrov

**Affiliations:** Faculty of Social Sciences, School of Economics and Business Administration, University of Tartu, Tartu, Estonia

**Keywords:** job satisfaction, cross-cultural studies, job demands, job resources, PIAAC dataset

## Abstract

This paper addresses whether supervisory responsibility is a challenging job demand in the Job Demands-Resources (JD-R) model in different cultural contexts. We investigate how job satisfaction responds to a supervisory role with job control and selected cultural dimensions using a cross-cultural dataset of 14 countries with more than 43,000 adults using ordered logit regression models. We find that a supervisory role enhances job satisfaction and appears to be a challenging job demand. However, no studied cultural dimension, masculinity, power distance, individualism, or uncertainty avoidance, increases job satisfaction derived from this kind of responsibility. Our study indicates that there might be stereotypical assumptions about cultural dimensions concerning the job satisfaction of supervisors.

## Introduction

There is a consensus that employee wellbeing necessitates sufficient resources that are required to perform one's job. This can be witnessed in the increasing popularity of the Job Demands-Resources (JD-R) model (Demerouti et al., 2001) in the last decade as a dominant theoretical framework (Jang et al., 2018; Rattrie et al., 2020). Job demands are defined as “physical, psychological, social, or organizational aspects of the job that require sustained physical and/or psychological effort and are therefore associated with certain physiological and/or psychological costs” (Bakker et al., 2010: 3). While the pool of potential demands has been expanded over the years, supervisory responsibility has not been conceptualized as the demand in the model.

Yet, supervisory responsibility offers an intriguing subject for research in the JD-R framework. Higher responsibility typically refers to higher task significance, which in turn is a job resource (Lee et al., 2020). The motivational effect may not be granted, however, because power may also embed aspects that the employee experiences negatively (Bless and Granato, 2018) and she would rather have “nothing to do with leadership responsibility or positional power as a resource within their role” (Lee at al., 2020: 26). The reasons may include role ambiguity, emotional and cognitive demands, higher workload, and possible health consequences in the long run (Johnston and Lee, 2013). In this case, supervisory responsibility is a demand, not a resource.

Furthermore, job demands can be motivating if these are of a challenging type (Crawford et al., 2010) as opposed to hindering type. However, there are no definite clusters for both. An individual may perceive certain demands as challenging and/or hindering. Given the evidence (Francesconi, 2001; Kosteas, 2011) that a managerial role is associated with higher job satisfaction, it presents a motivational effect in the JD-R model and would classify as challenging demand. Nevertheless, the supervisory role may be motivational in some contexts and not to the same extent in others. In this study, we focus on national culture as a boundary condition.

In the context of the JD-R model, national culture is essential due to several reasons. First, it determines the importance placed on a particular resource or demand, and second, it affects the effectiveness of specific features in achieving job outcomes (Farndale and Murrer, 2015; Jang et al., 2018; Prince et al., 2020). Furthermore, a manager's perception of their position may depend on national contextual factors (Hauff et al., 2015; Rattrie et al., 2020). The literature on motivational processes in the JD-R model based on cultural differences is scant (Jang et al., 2018). We theorize the effect of a supervisory role in different countries with a cross-cultural perspective to investigate the criticism of Western bias on the JD-R model (Verhoeven et al., 2003).

Hence, the objective of this study is to investigate the potential and limitation of supervisory responsibility in the JD-R model depending on national culture dimensions. Further, we investigate job satisfaction as a specific type of an employee's wellbeing (Hakanen et al., 2018) and an indicator of employee wellness (Verhoeven et al., 2003). We focus on the motivational process of the model rather than the health impairment or strain process (Bakker et al., 2014). We focus on job satisfaction because it leads to favorable organizational outcomes (such as commitment, endowing in firm-specific human capital, organizational citizenship behavior, and productivity). In the same vein, the lack of job satisfaction results in unfavorable outcomes (such as turnover, absenteeism, and workplace deviance) (Origo and Pagani, 2008; Chordiya et al., 2019). Thus, it is important to study job satisfaction as a significant attitudinal outcome and a channel for enhanced performance.

This study contributes primarily to the empirical literature on the JD-R model in relation to job satisfaction in a cross-cultural context. In contrast to previous studies, we explore supervisory responsibility, including direct and indirect subordinates. The novelty of this study is thus the examination of limitations in the JD-R model concerning supervisory responsibility in organizations.

Another contribution of this study is using data from the Program for the International Assessment of Adult Competencies (PIAAC), covering 22 countries. The PIAAC, a survey produced by the Organization for Economic Co-operation and Development (OECD), provides comprehensive information on adults' work-life and demographic characteristics. Even though the acknowledgment of potential cultural effects on JD-R predictions has increased (Fila et al., 2017; Saari et al., 2017; Jang et al., 2018; Rattrie et al., 2020), earlier studies addressing it have certain limitations. Saari et al. (2017) compared private-sector employees in two countries, Finland and Russia, which limits cross-cultural generalizations. Farndale and Murrer (2015) studied employees in different countries within one multinational corporation, where the influence of organizational culture could override national culture. Baba et al. (2013) studied nurses, and Chordiya et al. (2019) studied public sector employees, where both groups are inducted and socialized to solid professional values, which may transcend potential cultural influence. The PIAAC captures occupational and organizational variety; moreover, it is nationally representative, thus evading the typical generalizability problem in cross-cultural studies using convenience samples.

The next section presents a review of the literature to develop our hypotheses. Then we describe the dataset and discuss the research methods used. We present and discuss the results with study implications followed by limitations and future research opportunities.

## Hypotheses Development

The JD-R model determines the connection between job characteristics and employee wellbeing. In general, the job resources predict the motivational processes of the model, and the job demands predict the strain or health impairment processes (Bakker and Demerouti, 2017). Yet, the implications depend on the type of demand. While hindrance demands are perceived to interfere with the achievement of valued goals, challenge demands are understood to promote personal growth and achievement and are therefore seen as rewarding, despite the physiological and/or psychological costs (Crawford et al., 2010). Both types require matching resources to achieve optimum job outcomes (Madrid and Patterson, 2020; Van Veldhoven et al., 2020).

Empirical studies show that higher job status is positively associated with an employee's job satisfaction (Francesconi, 2001; Kosteas, 2011; Locke, 2020) and engagement (Saari et al., 2017). Even employees' optimism about getting a promotion within the next 2 years resulted in significantly higher job satisfaction (Johnston and Lee, 2013). Viñas-Bardolet et al. (2020) discovered that an opportunity for career advancement is *the* most important variable in explaining the European knowledge-workers' job satisfaction. A longitudinal study that investigated job satisfaction of two supervisor groups, designated and non-designated supervisors (those who had no formal title for that responsibility), reported persistently higher job satisfaction in the former group (Woodward et al., 2000). Thus, mere recognition as the manager is satisfaction enhancing, even if the content of the job is the same.

The above indicates that the supervisory role is satisfaction enhancing and, in general, a challenge demand. Studies suggest that a mix of reasonably high demands and high resources is ideal, while low demands and high resources are perceived as boring (Kwon and Kim, 2020). Following this, job satisfaction would be further enhanced by adding resources to the supervisory role. In our study, the matching resource would be job control. Job control is the most important job resource (Van Veldhoven et al., 2020) and an independent predictor of positive work outcomes, including job satisfaction (Bakker et al., 2010; Fila et al., 2017). Job control enables employees to engage in job crafting (Tims and Bakker, 2010), and in the presence of high job demands, such as supervisory responsibility, serves as an efficient way to maintain wellbeing. Hence, we hypothesize the following:

*H1a:* Supervisory responsibility has a positive effect on job satisfaction.
*H1b:* Interaction between supervisory responsibility and job control enhances job satisfaction.

“Management theories, such as the JD-R model, are influenced by national-level factors such as culture” (Farndale and Murrer, 2015: 611). One of the definitions of national culture in the work context is the “central organizing principle of employees' understanding of work, their approach to it, and how they expect to be treated” (Newman and Nollen, 1996: 755), and it naturally affects how people value and perceive incentives (Prince et al., 2020). Origo and Pagani (2008) noticed that the positive effect of work autonomy, a job resource, on job satisfaction is stronger for employees from Southern European countries compared with employees from Nordic, Central, and Anglo-Saxon regions. Sense of accomplishment had a stronger relationship with job satisfaction in masculine cultures of North- and Latin America (Jeanine et al., 2014), and managerial position predicts work engagement in Russia, but not Finland (Saari et al., 2017). In summary, the value of a managerial position and its effect on job satisfaction thereof may differ depending on the cultural context.

Building on previous cross-cultural JD-R research, we rely on Hofstede (1980) cultural dimensions, namely, masculinity (MAS), power distance (PD), individualism (IND), and uncertainty avoidance (UA). All the dimensions are expected to have implications for the supervisory role.

MAS denotes “a preference in society for achievement, heroism, assertiveness, and material rewards for success. Society at large is more competitive” (Hofstede, 2020, para. 10). MAS is a close counterpart to performance orientation, which is a cultural dimension identified in the GLOBE study (House et al., 2004). To theorize about the moderating effect of MAS or performance orientation, we emphasize that the basic role of a manager is to achieve goals through subordinates and is thus beyond the direct control of the incumbent. Yet having control is crucial for job satisfaction in high MAS cultures (Fila et al., 2017). In general, MAS amplifies the strain effect of job demands (Allen et al., 2020) and does not enhance the motivational effect of resources (Rattrie et al., 2020). Hauff et al. (2015) found no significant effect of MAS between advancement to higher-level jobs and job satisfaction. Therefore, it is likely that the managers in MAS cultures tend to experience less job satisfaction from their role, considering financial rewards and job control as constant. We propose the following hypothesis:

*H2:* MAS negatively moderates the relationship between supervisory responsibility and job satisfaction, such that the positive relationship is weaker when managers come from higher MAS cultures and stronger when managers come from lower MAS cultures.

PD refers to “the degree to which the less powerful members of a society accept and expect that power is distributed unequally” (Hofstede, 2020, para. 6). Supervising responsibility may be emotionally more fulfilling in high PD culture because power is accorded greater social value in the society (Locke, 2020). Indeed, Robie et al. (1998) and Locke (2020) found that managerial status in high PD cultures increased job satisfaction. Saari et al. (2017) conclude that managerial position contributes to work engagement in Russia and not in Finland exactly because the former has higher PD. However, Huang and Van De Vliert (2003) and Hauff et al. (2015) were unable to confirm their belief that advancement opportunities induce job satisfaction in high PD countries. Benson et al. (2020) later validated it by concluding that in high PD countries, people associated career success with security and satisfaction rather than performance and advancement. We hypothesize the following:

*H3:* PD positively moderates the relationship between supervisory responsibility and job satisfaction, such that the positive relationship is stronger when managers come from higher PD cultures and weaker when managers come from lower PD cultures.

IND is defined as a preference for a loosely-knit social framework in which individuals are expected to take care of only themselves and their immediate families (Hofstede, 2020, para. 8). IND might be favorable to the interaction between supervisory role and job satisfaction because managerial status is a sign of self-actualization, and “standing out from the crowd” is positively viewed in this culture. In contrast, job satisfaction is derived from group affiliation, maintaining harmonious relationships with peers, and stability in the collectivist culture (Yeh, 2015), which could be challenged by a managerial role. In individualist cultures, employees are expected to provide (critical) feedback to managers, whereas in collectivist cultures, diplomacy is valued. Managers in collectivist countries are more constrained by family ties and patronage in recruitment and advancement decisions (Chordiya et al., 2019). Feedback and independence as separate and important job resources (Lee et al., 2020) are less available for managers in collectivist cultures, and thus, job satisfaction may be affected. Studies have shown that decision latitude and job control are valued by individualist employees but not by collectivist employees. For the latter group, these are even additional stressors (Cendales and Gómez Ortiz, 2019).

Empirical research on JD-R model indicates that the relationship between job demands and strain tends to be stronger in IND cultures because promoting the self in the work domain competes with other spheres of life in IND cultures, whereas in collectivistic cultures, work is seen as contributing to family and community (Yang et al., 2012). However, unlike MAS and PD, studies have shown that IND significantly strengthens the positive effect of resources on work engagement (Prince et al., 2020; Rattrie et al., 2020). Both job demands and job resources tend to be more strongly related to job outcomes in IND cultures (Jang et al., 2018; Cendales and Gómez Ortiz, 2019; Allen et al., 2020). However, the moderating role of IND has been found to be insignificant in advancement opportunities (Huang and Van De Vliert, 2003; Hauff et al., 2015). The closest study on supervisory responsibility was conducted by Huang and Van De Vliert (2004), who found that job levels, including administrative and managerial tasks, had more influence on job satisfaction in IND cultures. Hence, we infer that IND leads to a positive perception of the supervisory role and amplifies its motivational effect. Therefore, we hypothesize the following:

*H4:* IND positively moderates the relationship between supervisory responsibility and job satisfaction, such that the positive relationship is stronger when managers come from higher IND cultures and weaker when managers come from lower IND cultures.

The UA dimension expresses the degree to which members of a society feel uncomfortable with uncertainty and ambiguity (Hofstede, 2020). Societies high on UA prefer having detailed rules, structured activities, and behavioral guidelines. Therefore, job resources to reduce uncertainty would be more valuable in higher UA cultures, and high UA would strengthen the positive effect of job resources. However, the empirical evidence is controversial. Jang et al. (2018) confirmed that UA positively moderates the relationship between job control and job satisfaction. In contrast, Hauff et al. (2015) and Rattrie et al. (2020) could not confirm any significant moderating effect of UA between job resources and work outcomes. Furthermore, Naseer et al. (2020) approached UA from a job-enrichment standpoint and confirmed that employees scoring high in UA perceived enriched jobs as a hindrance demand associated with negative outcomes.

Regarding supervisory responsibility, it is suggested that in high UA societies, advancement opportunities are less valued because enhancing one's career is not expected or socially desired (Smale et al., 2019). However, this suggestion is contested by a study that investigated the definition of career success of professionals in 15 countries, where respondents from high UA countries were more likely to define career success based on performance and advancement (Benson et al., 2020). We emphasize that it is more difficult for managers to plan their daily tasks in detail in terms of schedule and process; the job of a manager is inevitably more ambiguous compared with rank-and-file employees. Jeanine et al. (2014) concluded that managers working in high UA cultures should refrain from open communication with their employees. Jang et al. (2018) proposed that high UA limits the use of a job resource to reduce strain. Specifically, rules, detailed procedures, and regulations adopted by high UA societies limit the managers' chance to progress in their daily work. Hence, the discretionary power in being a manager is lower in a regulated environment and thus, contributes less to job satisfaction. We hence put forward:

*H5:* UA negatively moderates the relationship between increased supervisory responsibility and job satisfaction, such that the positive relationship is weaker when managers come from higher UA cultures and stronger when managers come from lower UA cultures.

**Figure 1:**
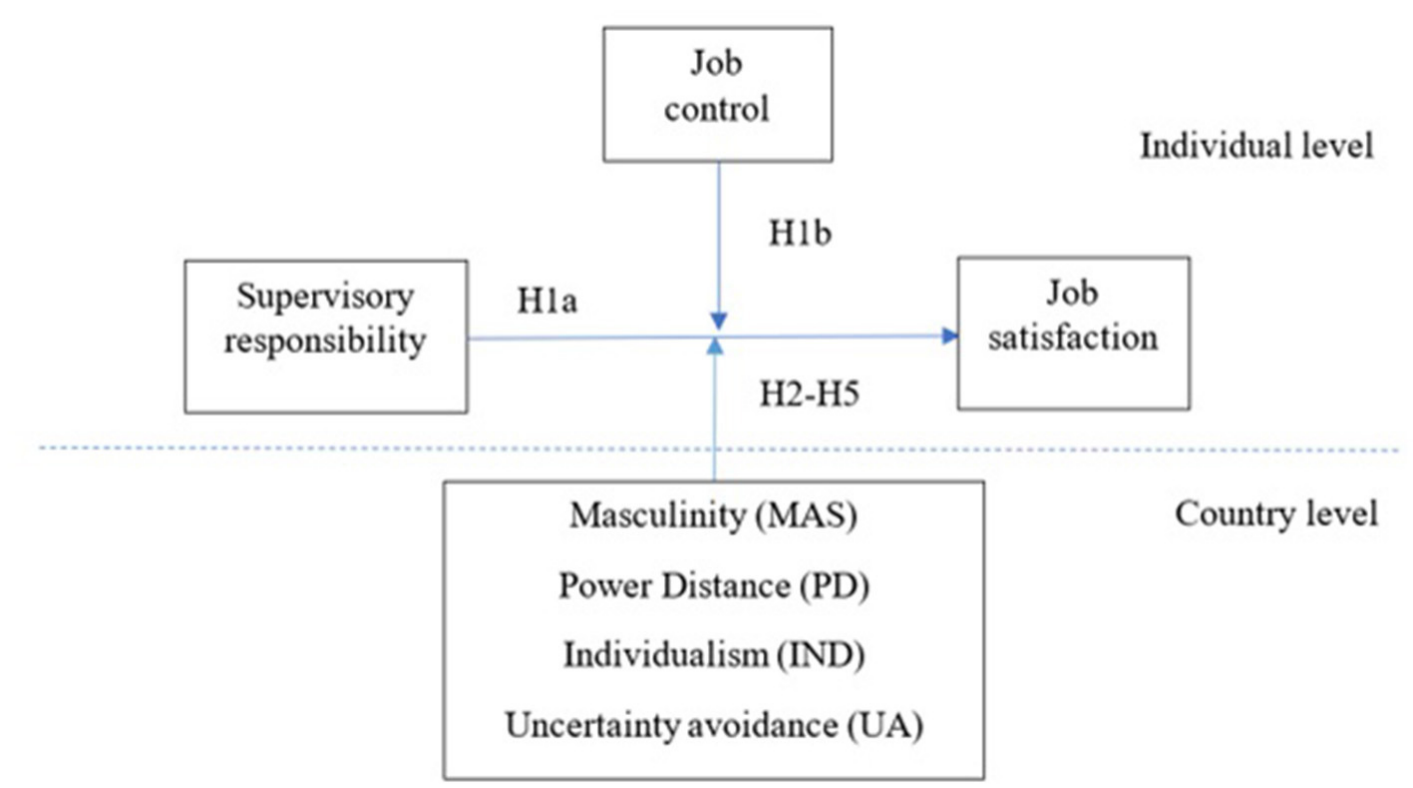
Study framework.

Our research framework is presented in Figure 1.

## Methods

### Data

This study uses the PIAAC dataset^1^ administered by the OECD between August 2011 and March 2012. PIAAC is conducted every 10 years and has had two cycles so far. Although PIAAC aims to measure the skills of adults in literacy, numeracy, and problem-solving in technology-rich environments, the survey also provides comprehensive job-related information. The responses for PIAAC were gathered from ≈166,000 adults who represented the population aged between 16 and 65 in 20 OECD countries, plus two non-OECD countries.^2^ Due to missing data for some items, especially pertaining to wages, cultural moderation estimations are based on 43,620 respondents from 14 countries. Participating countries were administered surveys in their national language, and comparability was established with rigid procedures and expert coding (OECD, 2013).

### Measures

#### Job Satisfaction

Our outcome variable is job satisfaction, with responses sought as follows: “All things considered, how satisfied are you with your current job?” Possible responses are: “Extremely satisfied,” “Satisfied,” “Neither satisfied nor dissatisfied,” “Dissatisfied,” and “Extremely dissatisfied.” As the two lowest categories (“Extremely dissatisfied” and “Dissatisfied”) contribute to <10 percent of the observations, these categories are merged into one.

#### Supervisory Responsibility

In the PIAAC questionnaire, the following question was asked from respondents: “Do you manage or supervise other employees? By managing or supervising other employees, we mean that a person is in some way responsible for how other employees do their work”. Supervisory responsibility is a dummy variable and takes a value of 1 if the respondent is supervising at least one subordinate and 0 otherwise.

#### Job Control

The variable reflecting how much control an employee has over their own work and the working environment is calculated as the mean of four items. The example questions are “To what extent can you choose or change the sequence of your tasks?” and “To what extent can you choose or change your working hours?” The scale ranged from 1 (not at all) to 5 (to a very high extent). Cronbach's alpha for job control was 0.816.

#### Cultural Dimensions

Scores for MAS, PD, IND, and UA were obtained from a publicly available database using European Social Survey and European Values Survey indicators, calculated by Kaasa et al. (2014)^3^ for 14 countries present in PIAAC. Indices of culture dimensions rely on large−scale surveys conducted in 2008, which is relatively close to the timing of the PIAAC survey. We concur with Jang etal. (2018) for using Hofstede's value dimensions instead of GLOBE or Schwartz (1992) values.

#### Controls

Employee-specific characteristics, particularly *age, tenure, health conditions, number of children, wage*, and *hours worked*, are vital factors that directly influence the wellbeing of a person. Another important characteristic is *gender;* while job demands and resources are positively related in male respondents, it tends to be negative in females (Fila et al., 2017). Another control variable is *the immigrant status* of the employee. The study by Chowhan et al. (2016) reveals that immigrants have lower job satisfaction than locals. We also establish control for the *company size* of the respondent's workplace (see Appendix for operationalization of control variables). Table 1 displays the correlations between control variables and job satisfaction.

**Table 1:** Means, standard errors and correlations between dependent and control variables.

**Variables**	**Mean**	**SE**	**1**	**2**	**3**	**4**	**5**	**6**	**7**	**8**	**9**
1. Job satisfaction	3.02	0.81	1.00								
2. Age	5.64	2.87	0.06^a^	1.00							
3. Health	2.60	1.07	−0.18^a^	0.23^a^	1.00						
4. Male	0.47	0.50	−0.01^a^	−0.01^a^	−0.03^a^	1.00					
5. Education	2.88	1.68	0.02^a^	−0.01	−0.10^a^	−0.09^a^	1.00				
6. Immigrant	0.14	0.34	−0.02^a^	0.00	0.01	−0.01	0.01^a^	1.00			
7. Relative wage (log)	1.18	0.89	0.07^a^	0.15^a^	−0.05^a^	0.25^a^	0.33^a^	−0.05^a^	1.00		
8. Working hours	19.77	21.39	−0.06^a^	0.06^a^	0.03^a^	0.27^a^	0.10^a^	−0.03^a^	0.40^a^	1.00	
9. No of children	1.30	1.25	0.08^a^	0.55^a^	0.11^a^	−0.05^a^	−0.03^a^	0.02^a^	0.09^a^	0.02^a^	1.00
10. Firm size	2.44	1.21	0.02^a^	0.05^a^	−0.05^a^	0.06^a^	0.18^a^	−0.01^a^	0.25^a^	0.10^a^	0.02^a^

*Authors' calculations. ^a^p < 0.01*.

### Estimation Method

This study employs the ordered logit estimation model with robust standard errors to examine if supervisory responsibility is associated with job satisfaction and the role of moderators in this link. The choice of model is justified because the job satisfaction variable is an ordered choice (categorical) variable. Alternative models, such as the logit regression for binary variables and OLS regression for continuous variables, are not suitable here. By using stepwise regression modeling, the first stage of analysis estimates the direct effect of the supervisory role on job satisfaction. The second stage of investigation looks at the challenging demand hypothesis by adding job control to the regression. The third stage will predict how country-level cultural dimensions and their interactions with individual-level variables are associated with job satisfaction. Furthermore, all cultural dimensions (MAS, PD, IND, UA) are standardized by using the sample mean and standard deviation (the process of subtracting the mean and dividing it by the standard deviation), and their z-scores are used in regressions. In models without cultural dimensions, country fixed effects are included. Further, an empirical analysis was performed using STATA 15.1 software.

## Results

First, we investigated if the supervisory role contributes to job satisfaction while holding the control variables fixed (see Table 2, Model 1). Being a supervisor contributes positively to job satisfaction, as expected in H1a (*b* = 0.17, *p* < 0.01; see Model 1). Model 2 tests if supervisory responsibility together with job control enhances job satisfaction. We find that the supervisory role coupled with job control is indeed positive and significant (*b* = 0.079, *p* < 0.01; see Model 2). Thus, H1a and H1b are confirmed, and we can conclude that the supervisory role can be deemed a challenging job demand and a positive contributor to job satisfaction by itself.

**Table 2:** Ordered logit estimations for job satisfaction.

	**Model 1**	**Model 2**	**Model 3**	**Model 4**	**Model 5**	**Model 6**
Individual level variables						
SR	0.174***	−0.193**	0.064***	0.0846**	0.0708***	0.0579**
	(7.78)	(−2.39)	(2.88)	(3.80)	(3.19)	(2.61)
Job control		0.306***	0.374***	0.326***	0.381***	0.333***
		(24.33)	(34.65)	(29.97)	(35.75)	(30.53)
SR * Job control		0.0794***				
		(3.37)				
Country level variables						
MAS			−0.160***			
			(−13.95)			
PD				−0.330***		
				(−28.89)		
IND					0.215***	
					(19.09)	
UA						−0.327***
						(−26.60)
Cross-level interactions						
SR * MAS			0.00562			
			(0.26)			
SR * PD				0.00170		
				(0.08)		
SR * IND					−0.0911***	
					(−4.50)	
SR * UA						0.0139
						(0.63)
Control variables	Yes	Yes	Yes	Yes	Yes	Yes
Country fixed effects	Yes	Yes	No	No	No	No
Observations	43,623	43,620	43,620	43,620	43,620	43,620
chi2	4,199.0	5,058.0	3,909.9	4,607.0	4,039.0	4,552.5
pr2	0.0476	0.0571	0.0422	0.0513	0.0437	0.0492
aic	92,556.9	91,627.4	93,053.9	92,171.1	92,910.4	92,375.1
bic	92,965.0	92,052.9	93,375.2	92,492.4	93,231.6	92,696.4

Source: Authors' own elaboration. Note: z statistics in parentheses. Robust standard errors are used. **p < 0.05, ***p < 0.01, SR, Supervisory responsibility for at least one employee; MAS, Masculinity; PD, Power distance; IND, Individualism; UA, Uncertainty avoidance.

Before moving on to cross-level interactions, we note that, in line with previous research, cultural dimensions themselves determine the level of job satisfaction. People are more satisfied with their jobs when located in higher IND countries and less satisfied with higher PD, MAS, and UA countries. In Models 3–6, we present the analysis of the moderating role of cultural dimensions. In H2, we suggested that job satisfaction decreases with supervisory responsibility in MAS cultures. Model 3 interaction term is positive (*b* = 0.005), but insignificant.

In H3, we proposed that the supervisory role has a stronger positive effect on job satisfaction in high PD cultures. Model 4 shows that the interaction effect is positive but insignificant (*b* = 0.0017, *p* > 0.05). Most surprisingly, the interaction coefficient for IND is *negative* and significant (*b* = −0.09, *p* < 0.01), which is opposite to H4. UA is insignificant, and hence H5 is not confirmed. Therefore, our results indicate that none of the cultural dimensions favor job satisfaction from supervisory responsibility.

### Robustness Test

Although empirical studies show that job satisfaction is more affected by the managerial yes/no status rather than the number of subordinates (Bless and Granato, 2018; Locke, 2020), it may be argued that supervising just one employee is not an appropriate indicator of supervisory responsibility. In the PIAAC study, the next threshold of supervisory responsibility status is “supervising five or more subordinates,” and when we operationalize the supervisory responsibility dummy as such, the direct effect on job satisfaction remains practically the same (*b* = 0.19, *p* < 0.01), see Table 3. Like in the previous model, job control enhances the managerial role's effect on job satisfaction (*b* = 0.09, *p* < 0.01). The moderating role of cultural dimensions changes on some occasions: for MAS, the interaction coefficient becomes negative but is still insignificant. For PD, the coefficient is *negative* and significant (*b* = −0.05, *p* > 0.05). IND remains negative but has become insignificant, similarly to UA. Hence, none of our hypotheses would hold under the described variable specification.

**Table 3:** Ordered logit estimations for job satisfaction with supervisory responsibility for at least five subordinates.

	**Outcome variable: job satisfaction**					
	**Model 1**	**Model 2**	**Model 3**	**Model 4**	**Model 5**	**Model 6**
Individual level variables						
SR(5)	0.190***	−0.248**	0.0645**	0.0648**	0.0528	0.0668**
	(7.21)	(−2.43)	(2.19)	(2.22)	(1.80)	(2.28)
Job control		0.302***	0.315***	0.368***	0.319***	0.363***
		(28.57)	(28.90)	(34.49)	(29.25)	(33.65)
SR(5) * Job control		0.0972***				
		(3.40)				
Country level variables						
MAS			−0.135***			
			(−12.69)			
PD				−0.315***		
				(−29.39)		
IND					0.188***	
					(17.99)	
UA						−0.313***
						(−27.31)
Cross level interactions						
SR(5) * MAS			−0.0294			
			(−1.01)			
SR(5) * PD				−0.0572**		
				(−2.09)		
SR(5) * IND					−0.0342	
					(−1.26)	
SR(5) * UA						−0.0434
						(−1.48)
Control variables	Yes	Yes	Yes	Yes	Yes	Yes
Country fixed effects	Yes	Yes	No	No	No	No
*N*	51,754	51,751	43,536	43,536	43,536	43,536
Chi square	6,518.2	7,514.6	5,119.8	4,546.0	5,050.0	4,403.0
Pseudo *R* squared	0.0603	0.0692	0.0572	0.0496	0.0552	0.0480
AIC	110,907.6	109,849.5	91,405.6	92,143.2	91,593.3	92,298.6
BIC	111,350.3	110,309.9	91,735.5	92,473.0	91,923.1	92,628.5

Source: Authors' own elaboration. z statistics in parentheses. Robust standard errors are used. SR(5), supervisory responsibility for more than five employees; MAS, masculinity; PD, power distance; IND, individualism; UA, uncertainty avoidance. ^**^p < 0.05, ^***^p < 0.01.

In many previous studies (Jang et al., 2018; Locke, 2020), cultural dimensions are retrieved from the Hofstede values database. In so doing, we could use all 17 countries in the PIAAC database. To test if our unexpected results may be caused by a different source of national culture values, we replaced Kaasa et al. (2014) values with Hofstede^4^ and ran our original models 3–6. The main effects of culture dimensions were similar to our previous findings, but all cross-level interaction coefficients were insignificant.

## Discussion and Implications

This study aimed to test the proposition that supervisory responsibility is a challenging demand in the JD-R model, and its motivational effect is dependent on the cultural context. Our contribution thus brought cultural context into the discussion on the JD-R model—a necessary stream of research (Jang et al., 2018)—using a large cross-cultural PIAAC database. Such a study is needed to address the criticism that management theories and models, such as JD-R, work only in Western contexts and not elsewhere (Verhoeven et al., 2003; Chordiya et al., 2019).

We conclude that supervising employees (not only processes or functions) is associated with job satisfaction, even though, similarly to earlier findings (Bless and Granato, 2018; Locke, 2020), the effect size is small. We argue that this is because supervisory responsibility operates like a challenging job demand in the JD-R model. Its motivational effect is pronounced if responsibility goes hand in hand with more job control, underlining the importance of balance between job resources and job demands (Bakker et al., 2010; Kwon and Kim, 2020; Madrid and Patterson, 2020). Practical implication stemming from our result is that, for one, resources should be available to support supervisors at times of additional stress. In our study, job satisfaction was enhanced by higher job control. Yet, job resources are not limited to control only. According to Lee et al. (2020), role (goal) clarity, task significance, and feedback are alternative job resources. Furthermore, other organizational, social, home or personal resources also deserve attention from managers and the company's human resources policies. Adding job demands without matching resources may become excessive and jeopardize the manager's wellbeing (Lochmann and Steger, 2002; Poelmans et al., 2003).

We expected that a more powerful role should be appreciated in high PD countries, but our results refute this intuition. Bless and Granato (2018) offer an interpretation that may be relevant for our study: initially, gaining a supervisory role brings about a positive affective experience, but in high PD societies, individuals start to strive for more power, which starts to decrease their job satisfaction. Another possible explanation is the more demanding nature of the managerial position in high PD cultures, whereby participation in decision-making and initiative is not expected from rank-and-file employees (Chordiya et al., 2019). Hence the responsibility falls on the shoulders of managers alone. Overall, the effects of demands are more straining in high PD cultures (Rattrie et al., 2020). The higher the PD, the less employees tend to experience a positive effect from high autonomy, high responsibility, or, generally, enriched jobs (Huang and Van De Vliert, 2003; Hui et al., 2004; Naseer et al., 2020).

Moreover, there might be fewer job resources available for managers in high PD cultures. For example, feedback provided to managers in high PD cultures is almost absent, and the manager's subjective feedback to employees is feared (Hwang and Francesco, 2010). Therefore, although respected by the subordinates, the manager lacks feedback, team support, subordinates' critical insight, and a sense of community. Finally, security is crucial in countries that emphasize hierarchy (Yeh, 2015; Benson et al., 2020), and the supervisory responsibility is more prone to the risk of redundancy. In high PD cultures, job satisfaction of managers should be monitored; supervisors in these cultures may benefit from coaching or mentoring that help the managers identify and use resources inside and outside the organization.

We expected that security is also more important to job satisfaction in MAS cultures (Hauff et al., 2015), and career advancement may come at the expense of security. In these cultures, the supervisory role may transform from a challenge demand to a hindrance demand more easily. MAS cultures are characterized by competition over cooperation, resulting in less social capital and trust (Kaasa, 2015), which, in turn, means managerial work may be more difficult to fulfill. In our study, we could not confirm that job demands (like supervisory responsibility) have stronger negative engagement effects in high MAS cultures (Rattrie et al., 2020) as the results were consistently insignificant. In sum, we cannot say that supervisors in these countries are “frustrated achievers,” as suggested by Graham and Pettinato (2002).

We expected that an IND cultural context would facilitate a supervisor's job satisfaction because challenging and interesting work is valued positively in IND culture (Hauff et al., 2015), and supervisory responsibility enabling this should be favorable to job satisfaction. Surprisingly, our results were contrary to expectations. We explain the finding by stressing that supervisory responsibility is a demand and not a resource in the JD-R model. Having said that, IND may amplify the strains associated with managerial work (Yang et al., 2012) or as put forward by Allen et al. (2020) p. 542: “demands from one role domain are more likely perceived as sources of conflict with the alternative role.” We suggest that while managers generally benefit from training programs on stress-management strategies and tools that help them to ground job demands (Lochmann and Steger, 2002) it is especially relevant in high IND cultures.

Finally, conforming to the general notion, higher UA does not enhance job satisfaction from the supervisory role, but no significant negative effect was found either. UA can be considered a litmus test for potential stress factors (Naseer et al., 2020), with challenge job demands easily transforming into hindrance demands for employees high in UA. Overall, our result conforms with recent meta-analyses—UA is an insignificant moderator between job demands and engagement (Rattrie et al., 2020) or job-satisfaction (Allen et al., 2020).

Therefore, an implication of our study is that the design of corporate incentive strategies may use stereotypical assumptions about cultural dimensions that should be revised. Assigning a supervisory role in high PD and IND environments maybe even detrimental to the person's job satisfaction in the long term. Based on the general notion that being a manager is reputable in high PD cultures, it does not, however, follow that highly respected managers are more satisfied as well. Similarly, high IND promotes employee job satisfaction in general but does not mean that supervisory responsibility is more satisfying in this environment.

## Limitations and Future Research

Regarding this study's limitations, PIAAC comprises self-reported data and standard caveats applied in this respect, including accuracy and social desirability bias. Due to cross-sectional nature of the data there are potential endogeneity issues between variables. We thus cannot claim for certain that the supervisory role increases job satisfaction, it may well be that more satisfied employees accept supervisory roles.

Job satisfaction was a single-item measure in the questionnaire, and its reliability may be criticized. However, this approach in JD-R research had been adopted by Demerouti et al. (2001), Farndale and Murrer (2015), Hauff et al. (2015), and Yeh (2015). Chordiya et al. (2019) argued in their study that in a cross-cultural context, a single-item measure is more comprehensive compared to a multiple-item. We nevertheless invite researchers to test cultural moderation with a multiple-item measure of job satisfaction. In addition, the supervisory role was defined by the pre-defined threshold categories with respect to the number of subordinates, which limits the use of PIAAC in studying the supervisory role in a more nuanced manner. Having the number of direct and indirect subordinates as continuous variables is rare but would be valuable in researching supervisors' wellbeing.

Cultural dimensions were assumed on a national level, though the dimensions may vary to a large extent within one country (Kaasa et al., 2014). Therefore, without more specific information on the region, assigning a general cultural dimension measure may be misleading for a particular respondent. In a study by Cendales and Gómez Ortiz (2019), there was a moderate but not perfect correlation between the national cultural dimension and its assessment by the individual. More configural approaches are needed to supplement country culture analysis (Allen et al., 2020).

PIAAC covers only OECD countries, therefore, we must be careful in making global generalizations. OECD countries are aging societies with labor shortage in many industries, making employers more willing to design jobs that ensures employees' job satisfaction.

Finally, our data is from 2011 to 2012, and drastic changes in the work environment have occurred since. Changes like digitalization and the gig economy have profoundly affected managerial work. Future studies should explore the second cycle of PIAAC data anticipated to be available by 2024.

## Data Availability Statement

Publicly available datasets were analyzed in this study. This data can be found here: https://www.oecd.org/skills/piaac/ and https://lepo.it.da.ut.ee/~akaasa/culturaldistances/datasources.html.

## Author Contributions

All authors listed have made a substantial, direct, and intellectual contribution to the work and approved it for publication.

## Funding

This work was supported by the Estonian Research Council grants PRG1513 and PRG791. Also, the support by the European Union Horizon 2020 research and innovation program grant agreement no. 822781 GROWINPRO is acknowledged.

## Conflict of Interest

The authors declare that the research was conducted in the absence of any commercial or financial relationships that could be construed as a potential conflict of interest.

## Publisher's Note

All claims expressed in this article are solely those of the authors and do not necessarily represent those of their affiliated organizations, or those of the publisher, the editors and the reviewers. Any product that may be evaluated in this article, or claim that may be made by its manufacturer, is not guaranteed or endorsed by the publisher.

## Footnotes

^1^More information can be obtained from https://www.oecd.org/skills/piaac/.

^2^Austria, Belgium, Canada, the Czech Republic, Denmark, Estonia, Finland, France, Germany, Ireland, Italy, Japan, Korea, the Netherlands, Norway, Poland, the Slovak Republic, Spain, Sweden, the United Kingdom (England and Northern Ireland), the United States, and the Russian Federation.

^3^Dataset obtained from: https://lepo.it.da.ut.ee/~akaasa/culturaldistances/datasources.html.

^4^Dataset obtained from Geert Hofstede website: https://geerthofstede.com/research-and-vsm/dimension-data-matrix/.

## Appendix

**Table A1:** Control variable descriptions in PIAAC database.

Age	Five year age intervals (16–19, 20–24, …, 55–59, 60–65)
Number of Children	Number of children, top-coded at 4 (0, 1, 2, 3, 4 or more)
Education	Education categories (1—secondary or below, …, 6—research master degree)
Health assessment	Health assessment: 1—“Excellent”, 2—“Very good”, 3—“Good”, 4—“Fair”, and 5—“Poor”
Immigration Status	Immigrant: 1—“1st or 2nd generation”, 0—otherwise
Gender	1—male, 0—female
Relative Wage	Relative earnings to country's median earnings
Working hours	current weekly working hours
Size of Company	Company size (1—up to 10 people, 2—11–50 people, 3—51–250 people, 4—251–1,000 people, and 5—more than 1,000 people)
